# The context of AI transformation: perceptions of AI ethicality and their impact on trust and use intentions

**DOI:** 10.3389/frai.2026.1841079

**Published:** 2026-09-16

**Authors:** Rachel Detherage, Shane Connelly

**Affiliations:** Department of Psychology, University of Oklahoma, Norman, OK, United States

**Keywords:** AI use, behavioral intentions, ethical AI, explainable AI (xAI), functionality, trust, proximity, situational factors

## Abstract

**Introduction:**

As the implementation of AI accelerates within organizations, concerns emerge about the ethicality of AI-driven systems and tools and the ways people use them. Although previous literature proposes numerous theories regarding how to develop and use AI ethically, questions remain about how users perceive the ethicality of AI and the effects this has on their engagement with AI tools. The study aims to explore whether and how certain perceptions of AI tool attributes, including beliefs about the explainability of the AI tool (xAI) and moral intensity dimensions (i.e., magnitude of consequences and proximity), influence perceptions of AI ethicality. Additionally, the study examines the effects of AI ethicality perceptions on the likelihood to recommend AI tools and functionality trust in AI.

**Methods:**

Researchers employ a randomized, controlled study design to investigate the relationships between the antecedents, perceptions of AI ethicality, and outcomes of functionality trust and behavioral intention.

**Results:**

Analyses revealed that AI ethicality perceptions significantly mediated the relationship between xAI and the likelihood to recommend the AI tool, whereas functionality trust in the AI tool significantly mediated the relationship between proximity and the likelihood to recommend the AI tool.

**Discussion:**

The results indicate the strength of users’ beliefs about xAI on their perceptions of AI ethicality and how users perceive AI as less capable of making decisions for human decision recipients. The perceptual nature of the study provides a theoretical contribution to the growing body of AI-related research relevant to organizations. The findings also offer a perspective to organizations implementing AI tools, signaling the need for management to consider context and ethical perceptions when implementing AI tools in the workplace.

## 1 Introduction

The growth of artificial intelligence (AI) in organizational spaces highlights how quickly the workplace can change and adapt to innovation. As new technologies surface, organizations use AI to transform commonplace work practices, increasing the accuracy and efficiency of various tasks and processes. Given such innovations, implementing AI in the workplace necessitates multiple standards to ensure its effectiveness, such as technological, legal, and ethical standards (Kelley, 2022). Ethical standards for AI in organizations safeguard employees, collaborators, customers, the organization, and other key stakeholders by requiring an AI tool to meet standards for implementation. In a recent study, Tossell et al. (2025) demonstrated AI’s ability to calibrate and recalibrate users’ moral judgment, emphasizing the importance of safeguards. However, such standards do not account for user perceptions of an AI tool as ethical or how such perceptions could affect their engagement with these tools (Khan et al., 2022; Mittelstadt, 2019). Employee perceptions of the ethicality and utility of organizational practices influence their broader views of the organization. For example, some research has shown that perceptions of AI ethicality in hiring practices impact organizational trust and organizational attractiveness for potential job seekers (Figueroa-Armijos et al., 2023; da Motta Veiga et al., 2023). The current study aims to employ a perceptual approach to understanding AI ethicality within organizations, as it remains a gap in the literature. AI ethicality requires a unique perspective due to the interdisciplinary nature of ethical dilemmas (i.e., computer science, philosophy, law, social sciences, etc.) surfacing with AI use (Biondi et al., 2023). The current study exhibits an interdisciplinary attitude by incorporating perspectives from AI development to reflect the computer sciences, descriptive ethics relevant to a philosophical viewpoint, the decision-making process as described by psychology, and applications paramount to business practices.

Artificial Intelligence (AI) refers to smart technologies with certain advanced capabilities generally performed by the human mind (Rai et al., 2019). As conceptualizations and practical applications of AI technologies continue to grow, questions arise about the ethical implications of using AI for predictions, recommendations, and the decisions stemming from its calculations. In a recent example of ethically questionable AI practices, racial and gender biases in AI-driven facial recognition software have surfaced (Coe and Atay, 2021; Lohr, 2022). Cases like this have emphasized the importance of reducing AI’s bias, especially against vulnerable populations (Farnós et al., 2025). Although the development of ethical AI allows systems to mitigate biases and other ethically problematic attributes, ambiguity remains about how the emerging workforce discerns what constitutes ethical AI. Situational characteristics, such as whether an AI tool exhibited intentionality, influence perceptions of blame when a decision causes harm (Sullivan and Fosso Wamba, 2022). The study model examines situational antecedents and whether the perceived ethicality of AI mediates between the antecedents and outcomes of functionality trust in AI and behavioral intentions through a randomized, controlled experimental design utilizing a scenario-based approach. This advances current knowledge about AI in the workplace by considering how various situational factors influence ethical perceptions of AI, as well as how those perceptions relate indirectly through perceived AI ethicality to key outcomes. By contributing to the growing research on AI and ethics within organizations, the model extends theory about AI ethics and advances the understanding of situational and perceptual factors regarding the implementation of AI tools in the workplace.

## 2 Literature review

Research examining ethical AI often focuses on its ethical development. Many AI tools in organizations use machine learning or computer algorithms that incorporate new data to adjust and improve predictions. As defined by Bleidorn and Hopwood (2019), machine learning refers to “the study and construction of algorithms that can learn from data and make predictions based on data without being programmed to perform specific tasks” (p. 191). This study uses the term AI to refer to machine learning tools, given the increasing development and use of machine learning tools and systems in organizations. For example, the ‘Human-in-the-Loop’ approach to AI development refers to the implementation of interaction between humans and AI during the AI data annotation, or training period, to improve machine learning in an ethical context. The process unfolds through stages (Chen et al., 2023). A set of training data consists of characteristics of an ethical situation (e.g., a profession’s code of ethics) with the ability to receive quantitative annotation. After the identification of a scenario, a human evaluates the same data as an AI tool by assigning a numerical value to each characteristic to depict its ethicality. The AI and human annotations undergo comparison, and the AI receives reinforcement through feedback. The AI continuously works with the human annotator until it produces annotations of the scenario aligning with real-world context (i.e., human judgments). This trains the AI to include ethical viewpoints in its decision-making calculus by incorporating a human viewpoint (Chen et al., 2023). However, as noted, this process does not necessarily result in ethical AI because human judgments can be biased, inaccurate, or unethical (Martin, 2019). The development of ethical AI tools must therefore address several concerns.

Common user concerns regarding AI include transparency, privacy, and fairness. In the context of AI, transparency exhibits how and why AI makes its decisions, whereas privacy refers to AI’s collection and regulation of personal data, and fairness contributes to AI’s ability to disregard discriminatory biases in its processes (Khan et al., 2022). Ethical AI requires contextual consideration beyond objective scientific principles, such as programming and training guidelines. Madanchian and Taherdoost (2025) highlight the normative and organizational importance of integrating ethics into AI adoption. Some theorists argue for a continuous, hybrid approach to AI use, where humans and AI work together in implementation to address some of the contextual pitfalls of AI technology (Billi and Labraña, 2025). Although the processes informing the development and implementation of AI tools are crucial to achieving ethical AI, understanding the factors influencing users’ ethical perceptions of AI tools can also offer insight into behavioral intentions and best practices.

### 2.1 The perceived ethicality of AI

The perceived ethicality of AI refers to how users evaluate an AI tool’s alignment with widely discussed ethical standards for AI tools. Although the perceived ethicality of AI could encompass a broad range of ethical principles, the current study references the ethical AI framework utilized by Jang et al. (2022), including dimensions of transparency, privacy, fairness, responsibility, and non-maleficence. The dimensions recur throughout research on ethical guidelines as prevalent and important themes (Fjeld et al., 2020; Jobin et al., 2019; Siau and Wang, 2020). Transparency refers to the ability of AI to disclose its technical procedures (Jang et al., 2022). Fairness encompasses the maintenance of an equitable and reasonable distribution of benefits and expenses without undue bias (European Commission, 2019). Privacy necessitates the ethical handling of data provided by the user and data generated about the user during their interaction with the system (European Commission, 2019). Responsibility requires the establishment of an accountable party for AI systems and their outputs during development, deployment, and usage (European Commission, 2019). Non-maleficence advocates for the development, implementation, and use of AI in a manner avoiding human harm (Floridi et al., 2018). The dimensions of the perceived ethicality of AI reference AI’s development, enactment, and usage to address ethical standards for the organizational implementation of an AI tool.

Jones (1991) utilizes the basis of Rest’s (1986) model of ethical decision-making to describe how situational factors influence ethical decisions. According to Rest’s (1986) model, ethical decision-making unfolds in four stages: moral sensitivity, moral judgment, moral intent, and moral behavior. The process begins with moral sensitivity, often called moral awareness, where an actor recognizes a moral issue, leading to a moral judgment, where the actor deciphers the moral correctness of an action. Moral intent then occurs, where the actor establishes the intent to act, resulting in the execution of the action during moral behavior (Jones, 1991; Rest, 1986). A decision about whether to use or trust AI may reference the ethical decision-making processes. Situational factors, as described by Jones (1991), can influence each stage of the ethical decision-making process, and existing literature examines the effects of situational influences on Rest’s (1986) stages (Lincoln and Holmes, 2011; Barnett and Valentine, 2004; McMahon and Harvey, 2007). Jones and Huber (1992) found that the combined effects of moral intensity predicted moral judgment, the stage of interest in the current study. Recent literature indicates AI-related contextual factors can influence relevant organizational outcomes. For example, autonomy-restricting AI increases unethical behavior in the workplace (Jolly et al., 2026).

### 2.2 Antecedents to the perceived ethicality of AI

Moral intensity theory refers to how the characteristics of moral issues affect the recognition of moral issues, development of moral judgments, establishment of moral intent, and engagement in moral behavior (Jones, 1991). Moral intensity shapes ethical decision-making, as different components impact its processes (Lincoln and Holmes, 2011). Situational factors, or dimensions of moral intensity, can influence how an individual views the ethicality of a situation. Jones’ (1991) model outlines six factors of moral intensity: magnitude of consequences, social consensus, probability of effect, temporal immediacy, proximity, and concentration of effect. Proximity and magnitude of consequences were chosen for the current efforts due to previously established theoretical and empirical relevance to the ethical decision-making stage of moral judgment (McMahon and Harvey, 2007; Morris and McDonald, 1995). Additionally, the experimental adaptability and parsimonious nature of the factors fit the current investigation. Other moral intensity factors, such as social consensus, could struggle with a scenario-based approach, whereas other factors face difficulty with operationalization, such as the probability of effect, given that the probability of harm from AI tools is uncertain.

The study also investigates xAI, or the extent to which an AI tool facilitates users’ understanding of the reasoning and processes leading to the tool’s results or decisions, as a situational factor. xAI acts as a situational factor because it affects users’ ability to understand information relevant to the decision-making process. Schwartz and Kusyk (2017) propose additional situational factors for the moral intensity framework, including issue complexity, defined as the degree to which a decision-maker perceives a moral issue as understandable or resolvable. xAI mirrors issue complexity, as users would inherently perceive an AI tool with an xAI description as more understandable. Often in AI tool development, developers will choose to take a “black box” approach for their model because it offers more accurate results, but the logic and decision-making processes of the model are unknown (Ali et al., 2023). xAI can build user trust and confidence in utilizing AI tools, which could affect perceptions of these tools and subsequent decisions to use them (Gutzwiller and Reeder, 2021; Hudon et al., 2021).

#### 2.2.1 Proximity as an antecedent to the perceived ethicality of AI

Proximity refers to the nearness a decision maker feels to the decision recipient (Jones, 1991). Definitions of proximity sometimes categorize proximity into different applications, such as social, psychological, and physical proximity or cognitive and emotional proximity (Mencl and May, 2009; Lee et al., 2018). Proximity exhibits an effect on moral awareness, reasoning, judgment, and intention in the ethical decision-making process (Lincoln and Holmes, 2011). Essentially, as proximity increases, individuals increase fairness and care within their ethical decisions (Wildermuth et al., 2017). Proximity also impacts an individual’s helping behaviors, implying that individuals feel more compelled to act ethically when close to those affected (Kogut et al., 2018). An increase in the moral awareness of a dilemma increases an individual’s sensitivity to their actions and the potential harm of those actions (Lincoln and Holmes, 2011). For example, when individuals feel another person falls within their scope of judgment, a harmful decision is judged as more unethical, illustrating proximity’s influence on the moral judgment (Singer and Singer, 1997). Proximity also affects how individuals compensate victims, supporting how psychological nearness to a situation brings the components of the ethical decision-making process to a more conscious level (Ghorbani et al., 2013).

Here, proximity applies in the psychological sense, where users feel a closer proximity to decisions directly affecting humans, as opposed to those affecting non-human decision recipients. For example, a decision about a dataset, such as whether to include a quarter’s earnings in a prediction model, might feel more distant and less proximal to the decision maker than a decision about whether to use an advanced algorithm to make hiring decisions affecting job applicants. Although the quarterly earnings data decision impacts individuals in the long term, the initial decision does not directly impact a human. Users report a greater psychological distance when working with AI agents compared to human agents, and users engaging with anthropomorphised AI tools feel psychologically closer to the AI agent compared to non-anthropomorphised AI agents (Ahn et al., 2021; Li and Sung, 2021). Although the study focuses on agents, rather than decision recipients, these findings support the idea of increased psychological proximity to humans in AI-related scenarios. Additionally, individuals make more principle or rule-based decisions in scenarios with closer proximity, supporting AI users’ choice to disregard the benefits provided by an AI tool in favor of preventing harm to human agents (Alfeo et al., 2026). Given proximity’s influence on the ethical decision-making process and users’ psychological distance from AI tools, the researchers hypothesize that decisions regarding human recipients will decrease user perceptions of AI ethicality.

*H1*: Users presented with a close proximity to the decision recipient will have lower perceptions of the ethicality of an AI tool.

#### 2.2.2 Magnitude of consequence as an antecedent to the perceived ethicality of AI

The magnitude of consequence refers to the sum of harms or benefits impacting decision recipients (Jones, 1991), including the number of people affected and the amount of harm or benefit. In application, the magnitude of consequence references the perceived harms or benefits of utilizing an AI tool in an organizational setting. Magnitude of consequences significantly influences the stages of the ethical decision-making process, including recognition of moral issues, moral judgments, and behavioral intentions (Barnett and Valentine, 2004; McMahon and Harvey, 2007; Lincoln and Holmes, 2011).

Similar to proximity, magnitude of consequences can align with components of the ethical decision-making process and the perceived ethicality of AI. For example, an AI system with a high, harmful magnitude of consequences, where its decisions could significantly, negatively impact individuals, may recalibrate a moral judgment, thus influencing judgments about the AI tool’s recommendations or predictions. The ever-expanding use of AI in the workplace presents a risk to users with a high potential for consequences. For example, users are less likely to support automating familiar tasks with significant impacts in the workplace (Horowitz et al., 2024). In recent research relating magnitude of consequences to AI tools, the perceived risk of an AI tool directly influences its perceived usefulness (Khan and Emon, 2024). Affecting ethicality, research has found a positive relationship between perceived benefits of AI use and ethical considerations (Bamigbola and Joseph, 2025). If a user perceives the AI tool as potentially harmful, then they would perceive the AI as less ethical. In operationalization, an AI tool making many decisions would have the ability to enact a large amount of harmful consequences, whereas an AI tool making few decisions would not have the opportunity to enact as much harm. Considering the influence of the magnitude of consequences on users’ ethical decisions, the researchers hypothesize that decisions with a lower indication of harmful consequences will present with increased perceptions of AI ethicality.

*H2*: Users presented with a low magnitude of harmful consequences will have higher perceptions of the ethicality of AI.

#### 2.2.3 xAI as an antecedent to the perceived ethicality of AI

Explainable AI (xAI) refers to the extent to which the data, algorithm, and decisions of an AI tool are explained and understandable (Ali et al., 2023). Though AI tools with an xAI feature require transparency from the AI about its training, processes, and decisions, xAI differs conceptually from transparency because the output must be functionally interpretable. With the growth in ethical development of AI, a variety of theories regard xAI as a component of ethical AI. For example, the Human-in-the-Loop system highlights the importance of transparency, fairness, and non-maleficence when developing AI tools (Chen et al., 2023). The intrinsic value of AI focuses on how xAI humanizes systems and supports user dignity (Colaner, 2022). Conceptually, xAI provides a window into the inner workings of the AI tool, alleviating concerns such as its ambiguity in how it arrives at its recommendations. For example, users rate AI tools with an xAI component as higher in fairness (Shulner-Tal et al., 2025).

AI tools with an explainable factor increase the AI’s perceived usefulness, perceived ease of use, trustworthiness, and trust intention for AI (Hamm et al., 2023; Meske and Bunde, 2023). If users have increased positive attitudes about AI, this could spill over into an increased perception of the ethicality of AI. Providing users with explanations (xAI) for AI decisions makes it more likely for them to take the advice, even when the advice is wrong (Van der Waa et al., 2021). This exemplifies how xAI may increase the likelihood of a user engaging with an AI tool because the perceptions of the ethicality increase, even without evidence of other ethical principles or outcomes. The current study considers xAI within user perceptions. Users do not interact with the AI tool, thereby conceptualizing users’ beliefs about xAI’s effect on ethicality, rather than a direct interaction between the user and xAI. Based on the connection shown in previous literature between xAI and positive attitudes toward AI, alongside xAI’s theoretical connection to transparency, the researchers hypothesize that AI tools described with an xAI element will increase users’ ethical perceptions of the AI tool.

*H3*: Users presented with xAI will have higher perceptions of the ethicality of AI.

### 2.3 Outcomes associated with perceived ethicality of AI

#### 2.3.1 Perceived ethicality of AI and likelihood to recommend an AI tool

Based on Rest’s (1986) model of ethical decision-making, making a moral judgment about a scenario precedes engaging in moral behavior. Applied to an AI context, users must judge an AI tool on its ethicality before deciding whether to recommend and/or use the tool. For example, individuals tend to choose between an AI decision-maker and a human decision-maker based on their evaluations of which is less biased (Pethig and Kroenung, 2023). If the AI tool seems biased to a user, it would be perceived as less ethical because biased technology violates ethical principles such as fairness and responsibility. This violation could lessen the likelihood of recommending or using the AI tool. Other ethical principles, such as privacy, have exhibited indirect effects on behavioral intentions regarding AI (Salam et al., 2026).

Other perceptual concepts, such as perceived usefulness, significantly affect behavioral intentions regarding AI tools (Ahn et al., 2021). Additionally, whether a user approaches AI with appreciation or avoidance influences their attitudes toward AI, impacting their behavioral intention (Cao et al., 2021). Specific to ethicality, users report higher perceived benefits of AI use when they also have higher expectations of an AI tool’s ethicality (Bamigbola and Joseph, 2025). Additionally, the domain where a user engages with an AI tool influences their likelihood of using AI, signaling the importance of ethical context in the calculus a user needs to make a judgment before engaging in a behavior (Maican et al., 2023; Schwesig et al., 2023; Logg et al., 2019). For example, Radanliev et al. (2024) discuss the heightened concern of privacy in sensitive domains such as healthcare. Positive ethical perceptions would increase a user’s behavioral intention to use an AI tool, whereas negative ethical perceptions would likely inhibit the use of the AI tool. Following the theoretical process of ethical decision-making, the researchers hypothesize that increased perceptions of ethicality will lead to greater behavioral intentions to recommend the AI tool.

*H4*: Users reporting higher perceptions of the ethicality of AI will show a greater likelihood of recommending an AI tool.

#### 2.3.2 Perceived ethicality of AI and functionality trust in AI

Mayer et al. (1995) define trust as an individual willing “to be vulnerable to the actions of another party based on the expectation that the other will perform a particular action important to the trustor, irrespective of the ability to monitor or control that other party” (p. 712). In a recent meta-analysis of human-AI trust, Vereschak et al. (2021) point out discrepancies within the research community for what constitutes trust in AI, and definitions are often incomplete or not included. Functionality trust refers to whether a user trusts the competency and expertise of an AI tool to perform a particular task (Choung et al., 2023). Choung et al. (2023) differentiate trust constructs by establishing human-like trust as trust in the agent (AI) and functionality trust as trust in the agent’s (AI’s) actions and abilities. Functionality trust in AI focuses on the AI tool’s ability to perform, including its competence and reliability, but does not include more human-like trust constructs, such as helpfulness or benevolence, which are often part of institutional trust in AI (Choung et al., 2023). Additionally, functionality trust does not encompass the trustworthiness of the organizations implementing the AI tool.

As previously stated, developers have increasingly added ethicality as a necessary component of AI. Therefore, ethicality appears as a built-in feature of technology. If the AI appears unethical, then the AI tool itself could be perceived as having performance deficits. Other abilities of AI, such as appearance and communication style, predict trust in AI (Kaplan et al., 2023). In the same way, the ethicality of an AI tool could be viewed as an ability of the AI, therefore also predicting functional trust in AI. If the AI tool’s recommendation violates an ethical principle, this changes the cognitive appraisal of the trustworthiness of the AI tool due to its decreased ability. Shah et al. (2024) provide evidence that users do not trust algorithms with perceived moral violations. Given the complementary nature of ethicality and functionality trust, the researchers hypothesize that increased ethical perceptions will lead to greater functionality trust.

*H5*: Users reporting higher perceptions of the ethicality of AI will show greater functionality trust in AI.

Functionality trust in AI may also directly influence a user’s behavioral intentions regarding the AI tool. In previous trust literature, trust has yielded effects on behavioral intentions such as risk-taking and task performance (Colquitt et al., 2007). Furthermore, trust in automated systems affects a user’s intention to engage with a system, their establishment of normative behaviors with the system, and their reliance on the system (Pavlou, 2003; Crisp and Jarvenpaa, 2013; Hoff and Bashir, 2015). Both human-like and functionality trust act as significant antecedents to attitude and perceived usage, resulting in a behavioral usage outcome (Choung et al., 2023). McGrath et al. (2025) showcased how trust in automation predicted intentions to rely on AI-generated recommendations. Relevant to perceived ethicality, Wang et al. (2025) found that trust in an AI chatbot significantly mediated the effects between perceived beneficence and the behavioral intention to recommend the AI chatbot. Considering the link connecting trust and behavioral intentions in past literature, the researchers hypothesize that greater functionality trust will increase users’ behavioral intentions regarding the AI tool.

*H6*: Users reporting a higher functionality trust in AI will show a greater likelihood of recommending the AI tool.

### 2.4 The model

Accordingly, the proposed model of perceived ethicality AI shown in Figure 1 anticipates relationships between the antecedents (proximity, magnitude of consequence, and xAI) and the outcomes (likelihood to recommend the use of an AI tool and functionality trust in AI), mediated by the perceived ethicality of AI. The model also explores functionality trust as a mediator between the perceived ethicality of AI and the likelihood to recommend the use of an AI tool. Figure 1 offers a visual representation of the model.

**Figure 1:**
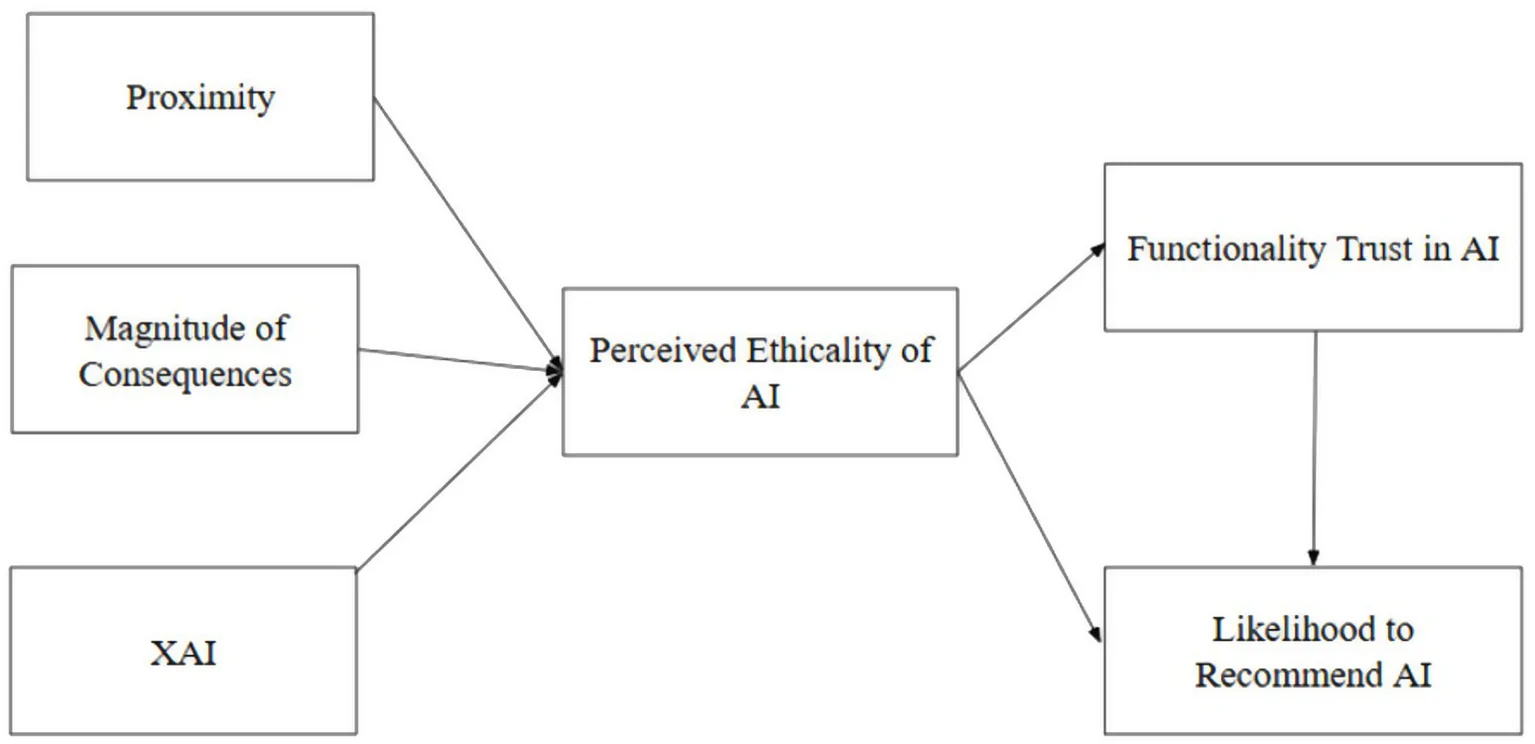
The concaptual model for antecedents and outcomes of the perceived ethicality of AI.

*H7*: The perceived ethicality of AI will mediate the relationships between the antecedents (proximity, magnitude of consequence, and xAI) and the outcomes (likelihood to recommend the use of an AI tool and functionality trust in AI). Antecedents and Outcomes of the Perceived Ethicality of AI.

## 3 Methodology

### 3.1 Study design and participants

A 2X2X2 between-subjects randomized, controlled study tested the hypotheses, with xAI assigned as present or absent, magnitude of consequences assigned as low or high, and proximity assigned as distal (low) or proximal (high). The study recruited volunteer participants from a large public university located in a southern state with a suburban setting to partake in the experiment. The proposed model is particularly relevant to the sample demographic, as students are currently facing the benefits and drawbacks of AI use in their education, an exemplar of an organizational setting (Klimova and Pikhart, 2025). Additionally, increases in age are associated with decreases in AI familiarity and the frequency of AI use (Chugunova et al., 2026). As students prepare to enter the workforce in an AI-advancing society, this sample demographic indicates the perceptions and behavioral intentions of the emerging workforce. However, students do not necessarily have experience making organizational decisions, nor are they expected to have a direct influence on AI implementation within the workplace, therefore limiting the representativeness of the sample.

Participant recruitment operated through the psychology department’s SONA online recruitment system, enabling students enrolled in psychology courses to earn class credit for participating in this study. All participants provided informed consent, and participants had to pass an attention check to ensure appropriate response behavior. A total of 572 participants completed the study within the data collection timeframe of 14 weeks. The sample included a slight female skew (74%), with other participants identifying as male (24.8%) or non-binary (0.7%), and 3 participants preferring not to identify (0.5%). The sample indicated a majority White/Caucasian racial demographic (64.3%), with other participants identifying as Hispanic (13.1%), Asian/Pacific Islander (8.2%), Black/African American (7.5%), Native American (4.5%), or other (2.3%), with the average age of the sample at 18.8 years (*SD*: 1.15).

### 3.2 Procedures

Participants completed the study using the Qualtrics online platform. Participants began by reading general background information about a fictitious organization, as well as their position and role within the organization. Next, participants were presented with three scenarios, each focusing on an AI tool under consideration for the organization. Appendix A contains the scenarios. For each participant, the manipulations remained constant across their 3 scenarios. After reading each scenario, participants continued by answering the perceived AI ethicality scale, adapted from Jang et al. (2022), relevant to the AI tool in the scenario, then the functionality trust scale for the AI tool, and a likelihood to recommend the tool for use in the organization, respectively. The scales can be found in Appendix B. Each participant evaluated three AI tools, following the procedure in its respective order (scenario, perceived ethicality, functionality trust, likelihood to recommend) before responding to manipulation checks and the covariate measures.

### 3.3 Manipulations

#### 3.3.1 xAI

Participants read scenarios manipulated by the presence or absence of xAI. Examples of the xAI manipulations: *(a) whenever you use the tool, the AI (will/will not) explain the rationale and processes it used to come to its decision; (b) if you use the AI tool, you (will/will not) be informed on how it was trained to make decisions*. The present xAI condition indicates the AI tool explains its training or rationale, whereas the absent condition denotes a lack of explainability.

#### 3.3.2 Magnitude of consequences

The scenarios included a magnitude of consequences manipulation of low or high. Examples of the magnitude of consequences manipulations: *(a) the organization is hiring (4/400) applicants from an applicant pool of (50/5000), (b) the decision impacts (one-twentieth/one-half) of the budget for marketing strategies.* The high condition signifies a higher stake for the decision, through the absolute number of decisions or the decision’s impact, whereas the low condition indicates a lower stake.

#### 3.3.3 Proximity

The scenarios utilized proximity manipulation of distal or proximal manipulation. Examples of the proximity manipulations: *(a) you are presented with an AI (content/hiring) tool that will increase the speed and accuracy of (content production/hiring candidates), (b) you are trying to determine (marketing trends/subordinates to send to training).* The proximal condition references a human recipient of the recommendationsmade by the AI tool, such as candidates or subordinates, whereas the distal condition references a non-human decision recipient, such as consequences for the company’s products.

### 3.4 Mediator and dependent variables

#### 3.4.1 Perceived ethicality of AI

The perceived ethicality of AI scale was adapted from the previously validated measure by Jang et al. (2022), contained 16 items with 3 items for fairness (α = 0.77), 3 items for transparency (α = 0.84), 3 items for non-maleficence (α = 0.65), 3 items for privacy (α = 0.87), 3 items for responsibility (α = 0.77), and 1 item for general ethicality. A 5-point Likert scale records answers from ‘*strongly disagree*’ to ‘*strongly agree*.’ Example questions for each domain include fairness: ‘the AI tool can make decisions with reasonable outcomes,’ transparency: ‘the AI tool discloses its technical process,’ non-maleficence: ‘the AI tool could be used in a way that would avoid harm,’ privacy: ‘the AI tool would handle the data it generates properly to protect privacy,’ responsibility: ‘the AI tool seems to have a party responsible for its implementation,’ and general ethicality ‘the AI tool is ethical.’ Responses to the questions for each of the 5 dimensions of ethicality were averaged across the 3 AI scenarios to create scores for each dimension. Scores across these dimensions were also averaged to generate an average score of overall perceived ethicality. The scale exhibited good reliability across scenarios (*α* = 0.89).

#### 3.4.2 Functionality trust in AI

The ‘Functionality Trust in Smart Technologies’ scale from Choung et al. (2023) measured functionality trust in AI. The scale underwent adaptation to reference the specific AI tool in the scenarios, rather than AI tools in general. The scale contains 5 items recorded on a 5-point Likert scale ranging from ‘*strongly disagree*’ to ‘*strongly agree*.’ Questions from the scale include ‘The AI tool has the features necessary to complete key tasks’ and ‘This AI tool is dependable,’ and the scale exhibited good reliability across scenarios (*α* = 0.89).

#### 3.4.3 Likelihood to recommend the AI tool

Participants completed questions on their likelihood to recommend the AI tool described in the scenario on a single item using a 6-point Likert scale from ‘*extremely unlikely*’ to ‘*extremely likely*’, with no neutral option.

### 3.5 Covariates

#### 3.5.1 AI self-efficacy

The AI self-efficacy scale from Wang and Chuang (2024) included 22 questions with 4 dimensions of assistance (*α* = 0.84), anthropomorphic interaction (*α* = 0.87), comfort with AI (*α* = 0.91), and technological skills (*α* = 0.70). A 7-point Likert scale recorded the answers from ‘*strongly disagree*’ to ‘*strongly agree*.’ Example questions include ‘AI technologies/products are good aids to learning,’ ‘*I think the interactive process of AI technologies/products is very vivid, just like chatting with a real perso*n,’ ‘When interacting with AI technologies/products, I find it easy,’ and ‘When using AI technologies/products I am not worried that I might press the wrong button and damage it.’ Overall, the scale exhibited excellent reliability (*α* = 0.91).

#### 3.5.2 Acceptance and use of technology

The acceptance and use of technology scale from Ustun et al. (2023) consisted of 18 questions with four subscales: performance expectancy (*α* = 0.86), social influence (*α* = 0.90), effort expectancy (*α* = 0.91), and facilitating conditions (*α* = 0.77). The scale utilizes unified technology acceptance and use of technology theory as developed by Venkatesh et al. (2003). The scale was adapted, so that ‘*artificial intelligence*’ replaced ‘*virtual reality.*’ A 5-point Likert scale recorded the answers from ‘*strongly disagree*’ to ‘*strongly agree*.’ Examples of questions include ‘Using artificial intelligence enables me to accomplish tasks more quickly’ and ‘My interaction with artificial intelligence is clear and understandable.’ The scale exhibited excellent reliability (*α* = 0.90).

#### 3.5.3 Personality

The Big Five Inventory (BFI-44), developed by John (1991), assessed participants’ personality attributes of extraversion (*α* = 0.86), agreeableness (*α* = 0.77), openness (*α* = 0.70), conscientiousness (*α* = 0.76), and neuroticism (*α* = 0.79). Participants rated on a 5-point Likert scale from ‘strongly disagree’ to ‘strongly agree’ statements of how they see themselves, such as ‘tends to be disorganized’ and ‘is full of energy’.

## 4 Results

After initial descriptive statistical analyses, inferential analyses included a series of ANCOVAs and mediation models. A summary of the Pearson’s *r* correlations can be found in Table 1 for the outcome variables and significant covariates included in the analyses, alongside scale reliabilities. Any covariate producing a significant zero-order correlation was included in the initial analyses but was dropped if it did not exhibit a significant effect in the model.

**Table 1:** Correlations of study outcomes and covariates.

Variable	*M*	SD	1.	2.	3.	4.	5.	6.	7.	8.	9.	10.	11.	12.	13.	14.
1. Perceived ethicality of the AI tool	3.06	0.45	(0.89)													
2. Functionality trust in the AI tool	3.28	0.61	0.66**	(0.89)												
3. Likelihood to Recommend the AI tool	3.29	1.03	0.63**	0.73**	(0.68)											
4. Neuroticism	3.16	0.70	−0.13**	−0.18**	−0.12**	(0.79)										
5. AI self-efficacy (Composition)	4.42	0.83	0.40**	0.47**	0.49**	−0.18**	(0.91)									
6. Assistance	5.11	0.99	0.36**	0.48**	0.48**	−0.17**	0.81**	(0.84)								
7. Anthropomorphic interaction	3.01	1.24	0.32**	0.31**	0.38**	−0.05	0.68**	0.38**	(0.87)							
8. Comfortability	4.86	1.09	0.33**	0.38**	0.39**	−0.18**	0.85**	0.62**	0.39**	(0.91)						
9. Technological skills	4.30	1.12	0.13**	0.17**	0.15**	−0.13**	0.64**	0.34**	0.26**	0.50**	(0.70)					
10. Acceptance and use of AI (Composition)	3.34	0.60	0.35**	0.44**	0.45**	−0.14**	0.73**	0.69**	0.45**	0.62**	0.39**	(0.90)				
11. Performance expectancy	3.35	0.79	0.31**	0.43**	0.46**	−0.10*	0.68**	0.71**	0.41**	0.56**	0.27**	0.87**	(0.86)			
12. Social interaction	3.01	0.95	0.30**	0.34**	0.39**	−0.07	0.51**	0.51**	0.35**	0.40**	0.22**	0.78**	0.61**	(0.90)		
13. Effort expectancy	3.85	0.68	0.21**	0.27**	0.24**	−0.13**	0.59**	0.48**	0.27**	0.59**	0.44**	0.74**	0.51**	0.37**	(0.91)	
14. Facilitating conditions	2.93	0.82	0.22**	0.21**	0.18**	−0.16**	0.32**	0.22**	0.29**	0.22**	0.23**	0.51**	0.24**	0.20**	0.32**	(0.77)

*Correlation is significant at the 0.05 level (2-tailed). ** Correlation is significant at the 0.01 level (2-tailed).

### 4.1 Manipulation checks

Participants completed manipulation checks at the end of the 3 scenarios. The proximity manipulation check asked participants if ‘the AI tools were used to make decisions directly about other humans,’ yielding significant differences between the distal (*M*: 3.03, SD: 0.92) and proximal (*M*: 3.86, SD: 0.78) manipulation [*t* (570) = −11.59, *p* < 0.001, *d* = −0.97]. The xAI manipulation check asked participants if ‘*it was difficult to know how the AI tools made their decisions*, yielding significant differences between the absent (*M*: 4.01, SD: 0.95) and present (*M*: 3.29, SD: 1.0) manipulation [*t* (570)=8.75, *p*<.001, *d* = 0.73]. The magnitude of consequences manipulation check asked participants, ‘*if recommended, the AI tools would have a large impact on the organization’s decisions*,’ yielding significant differences between the low (*M*: 3.64, SD: 0.89) and high (*M*: 3.93, SD: 0.78) manipulation [*t* (570) = −4.24, *p* < 0.001, *d* = −0.36].

### 4.2 ANCOVAs

A between-subjects full factorial ANCOVA indicated the main and interactive effects of the independent variables on the perceived ethicality of the AI tool, functionality trust in the AI tool, and the likelihood to recommend the AI tool. The ANCOVA for the perceived ethicality of the AI tool was overall significant [*F* (12, 559) = 21.1, *p* < 0.001, *R^2^* = 0.31], and xAI (M_absent_ = 2.90, *SD:* 0.43, M_present_ = 3.22, SD: 0.42) had a significant effect [*F* (1,559) = 89.6, *p* < 0.001, ηp^2^ = 0.14], whereas proximity and magnitude of consequence did not have significant effects. None of the two-way or three-way interactions were significant. These findings did not support Hypothesis 1 and Hypothesis 2, as neither proximity nor magnitude of consequences significantly, directly affect ethicality perceptions. However, the results do support Hypothesis 3, as the xAI manipulation increased user perceptions of ethicality. The ANCOVA for functionality trust in the AI tool was significant [*F* (11,558) = 23.68, *p* < 0.001, *R^2^* = 0.32]. xAI ( $M_{\text{present}}$ = 3.16, *SD:* 0.61, $M_{\text{absent}}$ = 3.4, *SD*: 0.58) had a significant positive effect [*F* (1,558) = 27.38, *p* < 0.001, ηp^2^ = 0.05] and proximity (M_distal_ = 3.33, *SD*: 0.61, M_proximal_ = 3.23, *SD*: 0.60) had a significant negative effect [*F* (1,558) = 7.21, *p* < 0.01, ηp^2^ = 0.01], whereas magnitude of consequence did not have a significant effect. None of the two-way or three-way interaction effects were significant. The ANCOVA for the likelihood to recommend the AI tool was also significant [*F* (11,560) = 29.9, *p* < 0.001, *R^2^* = 0.37]. xAI (M_absent_ = 3.02, *SD:* 0.98, M_present_ = 3.54, *SD:* 1.01) had a positive effect on likelihood to recommend the AI tool [*F* (1,560) = 48.47, *p* < 0.001, ηp^2^ = 0.08] and proximity (M_distal_ = 3.41, *SD:* 1.01, M_proximal_ = 3.17, SD: 1.03) had a significant negative effect [*F* (1,560) = 15, *p* < 0.001], whereas magnitude of consequence did not have a significant effect. None of the two-way or three-way interactions were significant. Table 2 summarizes the results of the three ANCOVAs.

**Table 2:** ANCOVA results for the effects of independent variables and covariates on study outcomes.

Source	Perceived ethicality of the AI tool			Functionality trust in the AI tool			Likelihood to recommend the AI tool		
	*f*	Sig.	ηp^2^	*f*	Sig.	ηp^2^	*f*	Sig.	ηp^2^
Corrected model	21.075	**<0.001**	0.311	23.676	**<0.001**	0.318	29.903	**<0.001**	0.37
Intercept	443.707	**<0.001**	0.443	111.465	**<0.001**	0.166	9.516	**0.002**	0.017
Covariates									
AI self-efficacy (assistance)	10.092	**0.002**	0.018	37.077	**<0.001**	0.062	24.867	**<0.001**	0.043
AI self-efficacy (anthropomorphic interaction)	12.314	**<0.001**	0.022	7.42	**0.007**	0.013	23.868	**<0.001**	0.041
AI self-efficacy (comfortability)	4.576	**0.033**	0.008	–	–	–	–	–	–
Acceptance and use of AI (social interaction)	7.48	**0.006**	0.013	–	–	–	7.222	**0.007**	0.013
Acceptance and use of AI (facilitating conditions)	6.92	**0.009**	0.012	–	–	–	–	–	–
Acceptance and use of AI (performance expectancy)	–	–	–	–	–	–	6.063	**0.014**	0.011
Acceptance and use of AI (composition)	–	**–**	–	10.82	**0.001**	0.019	–	–	–
Neuroticism	–	–	–	6.904	**0.009**	0.012	–	–	–
Independent variables									
Proximity	0.751	0.386	0.001	7.211	**0.007**	0.013	14.995	**<0.001**	0.026
Magnitude of Consequences	0.011	0.918	0	0.013	0.911	0	0.035	0.851	0
xAI	89.599	**<0.001**	0.138	27.377	**<0.001**	0.047	48.467	**<0.001**	0.08
Proximity * Magnitude of Consequences	0.028	0.867	0	0.557	0.456	0.001	0.749	0.387	0.001
Proximity * xAI	1.376	0.241	0.002	0.866	0.352	0.002	0.525	0.469	0.001
Magnitude of Consequences * xAI	0.405	0.525	0.001	1.366	0.243	0.002	1.133	0.288	0.002
Proximity * Magnitude of Consequences * xAI	0.007	0.934	0	1.5	0.221	0.003	0.007	0.932	0
*N* = 572	R Squared = 0.311 (Adjusted R Squared = 0.297)			R Squared = 0.318 (Adjusted R Squared = 0.305)			R Squared = 0.370 (Adjusted R Squared = 0.358)		

Bolded values indicate significance.

### 4.3 Mediation models

The PROCESS package version 4.2 tested the mediations through path analysis models, depicting the variables as outlined in the theoretical model to estimate the strength of direct and indirect relationships (main effects and interactions) for each manipulated variable (Hayes, 2017). For simple mediation, the model 4 iteration of the package estimated relationships, whereas the double mediation relied on the model 6 iteration of the package for estimation. Each analysis includes one independent variable, a mediator(s), and one outcome, as allowed by the package. For the indirect effects, the analyses utilized a bootstrapping method with 5,000 bootstrap samples. Three total models can summarize the findings. PROCESS model analyzes one predictor at a time, so the other two predictors (manipulations) were input as covariates in the model, which indicate the total effects of the predictors on the mediator(s) and outcome.

The first model employed a single mediation approach to analyze the effects of the predictors on the outcome of functionality trust through the perceived ethicality of the AI mediator. The combined predictors and covariates (acceptance and use of AI, AI self-efficacy, and neuroticism) had a significant direct total effect on the perceived ethicality of the AI tool [*F* (6,563) = 37.16, *p* < 0.001, *R^2^* = 0.28] and a significant direct total effect on the functionality trust of the AI tool [*F* (7,562) = 79.58, *p* < 0.001, *R^2^* = 0.50]. The second model employed a single mediation approach to analyze the effects of the predictors on outcome of likelihood to recommend through the mediator of perceived ethicality. The combined predictors and covariates (acceptance and use of AI, AI self-efficacy, and neuroticism) had a significant direct total effect on the perceived ethicality of the AI tool [*F* (6,564) = 37.16, *p* < 0.001, *R^2^* = 0.53] and a significant direct total effect on the likelihood to recommend the AI tool [*F* (7,563) = 76.28, *p* < 0.001, *R^2^* = 0.49]. The third model employed a double mediation approach to analyze the effects of the predictors on outcome on likelihood to recommend through two mediators: perceived ethicality of the AI tool, then functionality trust of the AI tool. The combined predictors and covariates (acceptance and use of AI, AI self-efficacy, and neuroticism) had a significant direct total effect on perceived ethicality [*F* (6,563) = 37.16, *p* < 0.001, *R^2^* = 0.53] a significant direct total effect on functionality trust [*F* (7,562) = 79.58, *p* < 0.001, *R^2^* = 0.50], and a significant total effect on likelihood to recommend when incorporating both mediators [*F* (8,561) = 104.80, *p* < 0.001, *R^2^* = 0.60]. Tables 3–5 report the pathways specific to proximity, magnitude of consequences, and xAI, respectively.

**Table 3:** Path coefficients and indirect effects mediation models with proximity as a predictor.

						95% CI	
Model	Path	*b*	*SE*	β	*t*	Lower	Upper
Proximity/PEAI/FTAI	PROX- > PEAI	−0.03	0.03	−0.06	−0.88	−0.09	0.04
	PROX- > FTAI	−0.08	0.04	−0.14	−2.27*	−0.15	−0.01
	PEAI- > FTAI	0.73	0.05	0.54	15.34***	0.64	0.82
	PROX- > PEAI- > FTAI *bootstrapped*	−0.02	0.02			−0.07	0.02
Proximity/PEAI/LTR	PROX- > PEAI	−0.03	0.03	−0.06	−0.88	−0.09	0.04
	PROX- > LTR	−0.27	0.06	−0.22	−3.65***	−0.35	−0.10
	PEAI- > LTR	1.08	0.08	0.48	13.32***	0.92	1.24
	PROX- > PEAI- > LTR *bootstrapped*	−0.03	0.04			−0.10	0.04
Proximity/PEAI/FTAI/LTR	PROX- > PEAI	−0.03	0.03	−0.06	−0.88	−0.09	0.04
	PROX- > FTAI	−0.08	0.04	−0.14	−2.27*	−0.15	−0.01
	PROX- > LTR	−0.16	0.06	−0.16	−2.92**	−0.27	−0.05
	PEAI- > FTAI	0.73	0.05	0.54	15.34***	0.64	0.82
	PEAI- > LTR	0.50	0.09	0.22	5.82***	0.33	0.67
	FTAI- > LTR	0.8	0.06	0.47	12.55***	0.33	0.67
	Total Indirect Effects	−0.09	0.04			−0.18	−0.01
	PROX- > PEAI- > LTR *bootstrapped*	−0.01	0.02			−0.05	0.02
	PROX- > FTAI- > LTR *bootstrapped*	−0.06	0.03			−0.12	−0.01
	PROX- > PEAI- > FTAI- > LTR	−0.02	0.02			−0.05	0.02

*p < 0.05; **p < 0.01; ***p < 0.001. PROX, proximity; PEAI, Perceived ethicality of AI; LTR, Likelihood to recommend; FTAI, Functionality trust in AI. Covariates included in the model: Acceptance and Use of AI, AI Self-Efficacy, Neuroticism, and other predictors (MOC and xAI).

**Table 4:** Path coefficients and indirect effects mediation models with magnitude of consequences as a predictor.

						95% CI	
Model	Path	*b*	SE	*β*	*t*	Lower	Upper
MOC/PEAI/FTAI	MOC- > PEAI	0.01	0.03	0.03	0.44	−0.05	0.08
	MOC- > FTAI	0.00	0.04	0.00	0.07	−0.07	0.07
	PEAI- > FTAI	0.73	0.05	0.54	15.34***	0.64	0.82
	MOC- > PEAI- > FTAI *bootstrapped*	0.01	0.02			−0.04	0.06
MOC/PEAI/LTR	MOC- > PEAI	0.01	0.03	0.03	0.44	−0.05	0.08
	MOC- > LTR	0.00	0.06	0.00	0.07	−0.12	0.13
	PEAI- > LTR	1.08	0.08	0.48	13.32***	0.92	1.24
	MOC- > PEAI- > LTR *bootstrapped*	0.02	0.04			−0.05	0.08

*p < 0.05; **p < 0.01; ***p < 0.001. MOC, Magnitude of consequences; PEAI, Perceived ethicality of AI; LTR, Likelihood to recommend; FTAI, Functionality trust in AI. Covariates included in the model: Acceptance and Use of AI, AI Self-Efficacy, Neuroticism, and other predictors (PROX and xAI).

**Table 5:** Path coefficients and indirect effects mediation models with xAI as a predictor.

						95% CI	
Model	Path	*b*	*SE*	β	*t*	Lower	Upper
XAI/PEAI/FTAI	XAI- > PEAI	0.31	0.03	0.68	9.55***	0.25	0.37
	XAI- > FTAI	0.01	0.04	0.01	0.13	−0.07	0.08
	PEAI- > FTAI	0.73	0.05	0.54	15.34***	0.64	0.82
	XAI- > PEAI- > FTAI *bootstrapped*	0.22	0.03			0.17	0.29
XAI/PEAI/LTR	XAI- > PEAI	0.31	0.03	0.68	9.55***	0.25	0.37
	XAI- > LTR	0.16	0.07	0.16	2.45*	0.03	0.30
	PEAI- > LTR	1.08	0.08	0.48	13.32***	0.92	1.24
	XAI- > PEAI- > LTR *bootstrapped*	0.33	0.04			0.25	0.42
XAI/PEAI/FTAI/LTR	XAI- > PEAI	0.31	0.03	0.68	9.55***	0.25	0.37
	XAI- > FTAI	0.01	0.04	0.01	0.13	−0.07	0.08
	XAI- > LTR	0.16	0.06	0.16	2.69**	0.04	0.28
	PEAI- > FTAI	0.73	0.05	0.54	15.34***	0.64	0.82
	PEAI- > LTR	0.50	0.09	0.22	5.82***	0.33	0.67
	FTAI- > LTR	0.8	0.06	0.47	12.55***	0.33	0.67
	Total Indirect Effects	0.34	0.05			0.24	0.44
	XAI- > PEAI- > LTR *bootstrapped*	0.15	0.04			0.09	0.22
	XAI- > FTAI- > LTR *bootstrapped*	0.00	0.03			−0.06	0.06
	XAI- > PEAI- > FTAI- > LTR *bootstrapped*	0.18	0.03			0.13	0.24

*p < 0.05; **p < 0.01; ***p < 0.001. XAI, Explainable AI; PEAI, Perceived ethicality of AI; LTR, Likelihood to recommend; FTAI, Functionality trust in AI. Covariates included in the model: Acceptance and Use of AI, AI Self-Efficacy, Neuroticism, and other predictors (PROX and MOC).

As seen in the second mediation model across manipulations, the perceived ethicality of the AI tool exhibited a significant pathway to users’ likelihood of recommending the AI tool, supporting Hypothesis 4. Additionally, the significant pathways between perceived ethicality and functionality trust support Hypothesis 5, and the double-mediation models provide support for Hypothesis 6 with significant pathways between functionality trust and likelihood to recommend the AI tool. Perceived ethicality significantly mediates the relationship between xAI and the study outcomes, but the other manipulations (i.e., proximity and magnitude of consequences) do not show significant pathways to perceived ethicality, thereby only partially supporting Hypothesis 7. Figure 2 summarizes the empirical pathways of the model.

**Figure 2:**
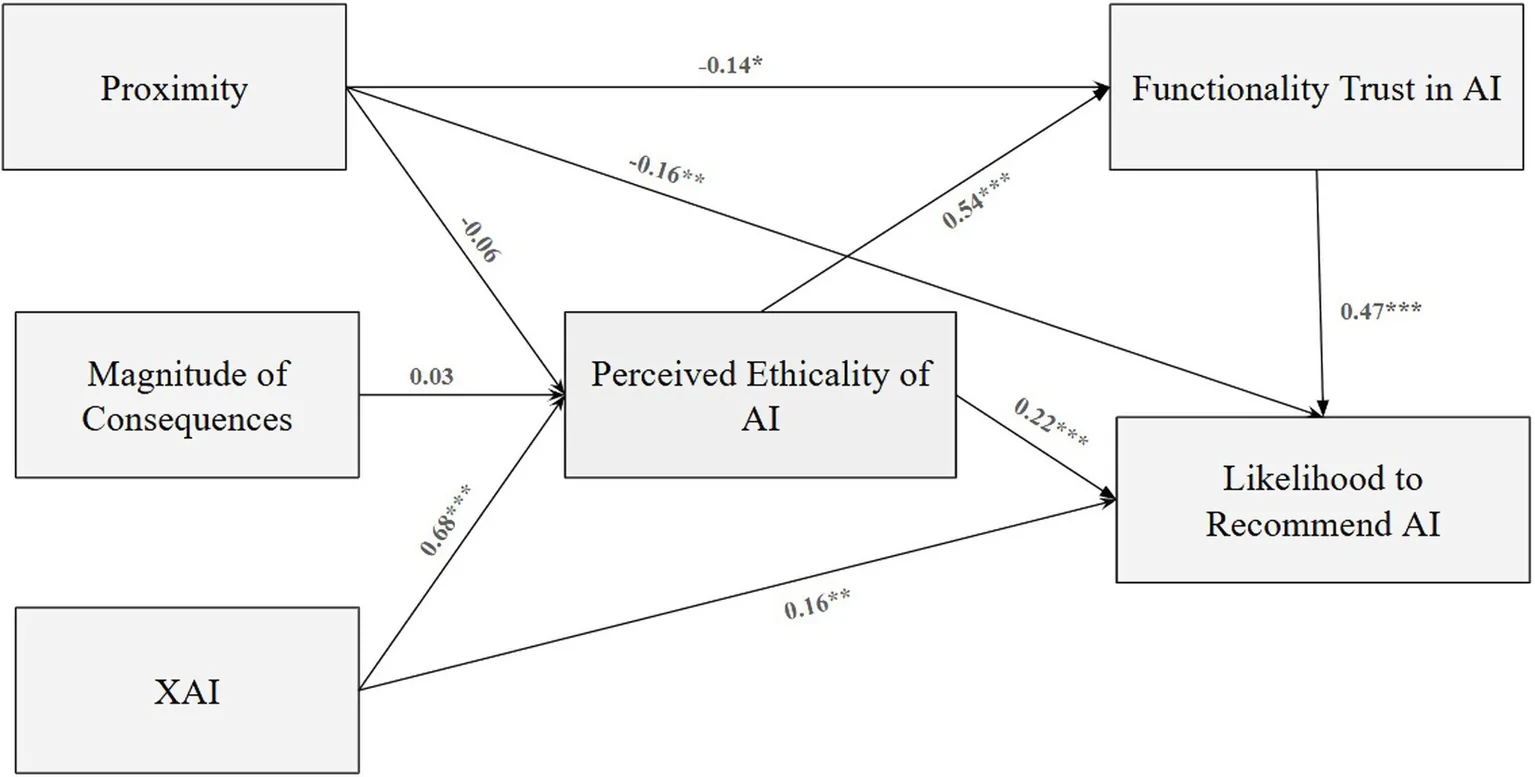
The path model for antecedents and outcomes of the perceived ethicality of AI. *p<0.05; **p<0.01; ***p<0.001.

Pathways for the Antecedents and Outcomes of the Perceived Ethicality of AI.

## 5 Discussion

Ethical AI theories emphasize the importance of ethical development and use of AI tools, but do not consider user perceptions of ethicality. This study examined how situational ethical factors influence user perceptions, trust, and intentions to use AI tools. The contributions of the research include the consideration of perceptual factors relevant to ethical AI, linking users’ ethical perceptions to trust and use outcomes, and employing an experimental approach to understand causal influences of AI features on ethical perceptions and other outcomes.

Regarding the covariates, users who viewed AI as more helpful and human-like showed significantly higher scores on all three outcomes, and users with higher comfort toward AI rated the AI tools as more ethical. Wang and Chuang (2024) discuss how AI self-efficacy enables users to feel more confident about AI tools. This confidence could connect to user confidence in its properties, such as its ethicality and capability, and whether the user would feel confident recommending the AI tool. Users experiencing strong social influence rated the AI tools as more ethical and were more likely to recommend their use. If users feel normative pressure to use AI, they may increase their behavioral intentions (Venkatesh et al., 2003). Additionally, users experiencing normative pressure to use AI may also experience norms including positive perceptions of AI tools, thereby impacting their ethical perceptions. Users with more resources and support in understanding and using AI (i.e., the facilitating conditions subdimension) also rated the AI tools as more ethical, possibly due to a better understanding of the collaboration between AI tools and users. Users expecting higher performance from the AI were more likely to recommend the AI tool, as their decision calculus would include increased benefits of using the AI tool. Overall, users with higher acceptance and use of AI had higher trust in the functionality of the AI tools. Users higher in neuroticism rated the AI tool as less functionally capable, as they may feel more nervous about trusting AI tools with human-like functions.

In the PROCESS models, proximity exhibited a significant effect on a user’s likelihood to recommend the AI tool alongside their functionality trust but did not show a significant mediation through the perceived ethicality of AI. Participants functionally trusted and recommended AI tools less if the decision directly influenced another person, which has the theoretical underpinnings of ethicality, thereby affecting their likelihood of recommending the AI tool in the organization. As seen in Martin and Waldman (2023), users can perceive lower legitimacy for AI decision makers when the decisions hold higher importance. Proximity may signal to the user that the decision has higher importance, resulting in less trust in its capabilities.

The magnitude of consequences did not show any significant main effects. The lack of significant results could relate to the findings of the proximity manipulation. Proximity may inherently raise the importance of the scenarios for users making proximal decisions. Users could attend more to the proximity information to infer the level of perceived consequences, placing less emphasis on the manipulation of the percentage of decisions the AI tool made. Additionally, the consequences of the AI manipulation may have left room for additional interpretation of potential consequences due to the broad, interdisciplinary effects of AI use. Some users could consider consequences pertaining to decision outcomes, such as who receives a job offer or what product receives higher ratings, whereas other users consider consequences pertaining to using AI tools in the workplace in general, such as precedence or job security. For example, societal consequences may be seen as more pertinent than described in Jones’ (1991) model. Regarding the operationalization of the magnitude of consequences, the study exercised a relatively narrow application, focusing on the total stakes or the importance of the decision. A more well-rounded operationalization could include the severity or distribution of the consequences, and further research should expand the operationalization to incorporate a more holistic quantification of consequences, as this could better encapsulate the construct in its entirety.

The perceived ethicality of the AI tool significantly mediated user beliefs about xAI and user likelihood to recommend the AI tool, whereas functionality trust in the AI tool did not. Perceptions of xAI influenced users to see AI as more ethical overall. Users’ perceptions of xAI play an important role by impacting perceptions of the ethicality and user intentions toward AI tools. Theoretically, an AI tool with a perceived explainable factor can inform users whether the AI tool operates in a fair manner, protects user privacy, takes relevant situational factors into consideration, or recognizes potential negative consequences. A perception of increased access to information can augment rational agency, referencing a user’s ability to integrate available information into their decision calculus (Feltz and Cokely, 2024). Therefore, the perceived presence of xAI may calibrate moral judgments by offering more information. However, explainability in an AI tool only offers meaningful information to users if the information is discernible. The information may only be provided in a technical form, such as lines of code about how the tool processes input. Even if an AI tool contains explainable features, this may not hold practical importance to everyday users without a technical background. Thereby, the significant results of xAI’s impact on perceived ethicality and usage intentions remain hypothetical, depending on the practicality and accessibility of the information provided to users. Additionally, national cultural factors could play into the interpretation and effects of xAI. For instance, uncertainty avoidance, the degree of which a culture tolerates ambiguity (Hofstede, 2001), could affect how explainability influences ethical perceptions, as cultures with a high uncertainty avoidance may show stronger effects of xAI.

### 5.1 Implications

#### 5.1.1 Theoretical implications

The findings of the study highlight how situational information shapes ethical perceptions and subsequent trust or behavioral intentions when considering the use of AI tools in an organizational setting. The findings use the moral intensity framework by Jones (1991) as a reference, applying moral intensity to decision-making in an AI context, focusing on the moral judgment stage of decision-making, as outlined by Rest’s (1986) model. However, the model may not fully encapsulate the broad, interdisciplinary nature of AI’s effects and how users consider these effects. Though theoretical frameworks highlight the ethical benefits of adding xAI to AI tools, the results of this study indicate a possible downside to descriptions of xAI attributes. Similar to the findings of van der Waa et al. (2021), AI users without a complex understanding of AI may overestimate the implications of AI tools with an xAI feature. A description of explainable features can influence users to perceive an AI tool as more ethical, thereby increasing trust in the AI tool, though users may not necessarily have the technical skills to evaluate the tool.

The basis of the research also expands the current ethical theories about AI use by incorporating user perceptions of ethicality. Though AI tools necessitate ethical development, user perceptions of AI ethicality require proper calibration to ensure ethical use. The study expands Jang et al.’s (2022) framework of AI ethicality by adapting the scale to account for specific instances of AI use, rather than broad attitudes. General attitudes about AI ethicality may provide less utility for an organizational situation as opposed to the perceived ethicality of the AI. Before employing any intervention to enhance user understanding of AI ethicality, researchers must comprehend the baseline perceptions of users. Though the study aims to begin the investigation, other situational factors can affect perceptions of AI ethicality, thereby warranting further evaluation. The current research can be integrated into theories about ethical AI by elaborating on user perceptions of ethical constructs.

#### 5.1.2 Practical implications

The results align with ongoing xAI research supporting the implementation of xAI features in AI tools. However, an AI tool may explain its training process or algorithmic weights, but this does not necessarily indicate other ethical attributes (i.e., fairness, nonmaleficence, etc.), misinforming AI users of its composite ethicality. The addition of xAI does not constitute ethical AI itself, though it heavily influences perceived ethicality. Organizations implementing AI tools should consider employee perceptions of the AI’s ethicality and how these may not reflect objective ethical standards. This is especially relevant when organizations consider guidelines and AI governance policies for the ethical development and implementation of AI. Organizations may want to include ethical perceptions of an AI tool as a standard for its implementation. If an AI tool does not perceptually meet the ethical standards for AI implementation, then the organization has the responsibility to address the ethical concerns of its employees. For instance, increasing the explainability of AI tools can offer employees more information about AI implementation. Given the unknown long-term consequences of implementing AI in organizational settings, organizations could see positive results from more ethically conservative implementations (e.g., decisions with non-human decision recipients or AI tools with explainable features), such as increased trust or behavioral outcomes.

### 5.2 Limitations

The design and analyses conducted in the study provide pertinent information to the research area but are not without limitations. The study utilized the descriptions of AI attributes in the scenarios, so the decisions may not entirely generalize to user interactions with AI tools, as the skills and knowledge necessary to comprehend and use xAI features are not expected among the sample. The study also implemented a scenario-based approach, measuring self-reported behavioral intentions, rather than observed behavior. The scenarios follow the decision points discussed by Aguinis and Bradley (2014), but the methodology may not fully generalize to observed behaviors. Future designs should supplement the results from this study by conducting research with direct measures of user behavior, thereby accounting for possible variation between behavioral intentions and behavior. The methodology also presents the independent variables within a dichotomy, but the factors can exist along a continuum. For example, some AI tools may have partial explainability, and decisions can have an array of consequences. Additionally, the study does not account for additional contextual factors, such as the organizational factors that may influence study variables. Factors such as organizational climate and culture, leadership, and group dynamics could be relevant to perceptions of AI in the workplace and need further exploration to contextualize AI use behaviors.

### 5.3 Conclusion

AI’s increasing implementation in organizational settings warrants further investigation of user perceptions and behavioral intentions. The study aims to understand how ethical perceptions stem from situational factors, thereby affecting trust and use intentions for AI tools. User beliefs about xAI exhibit a strong effect on the perceived ethicality of an AI tool, influencing how users trust and intend to use the AI tool. The model also found support for functionality trust mediating proximity and behavioral intentions to use the AI tool. Given the findings, the implications focus on the insight brought by understanding the effects of situational factors, alongside how organizations can leverage these factors to positively influence user perceptions, trust, and use. Future directions include measuring direct behaviors and expanding the design to incorporate more diverse situational and contextual factors.

## Data Availability

The datasets presented in this study can be found in online repositories. The names of the repository/repositories and accession number(s) can be found at: https://doi.org/10.17605/OSF.IO/GZFNY.
