# Supplementary material for: The context of AI transformation: perceptions of AI ethicality and their impact on trust and use intentions
Source: Front Artif Intell. 2026 Sep 16;9:1841079. doi: 10.3389/frai.2026.1841079 (PMC13624155; doi:10.3389/frai.2026.1841079)
Supplement: Supplementary file 1 [file Supplementary_file_1.pdf]

Appendix A

Scenario 1: You are presented with an AI tool that helps make (hiring/content) decisions. The AI tool will choose (candidates to hire/content to create) based on current market and organizational data. Whenever you use the tool, the AI (offers/does not offer) access to the datasets from its development. This means you (can/cannot) understand how the AI tool was developed. The tool will be utilized for (5 out of the 100/50 out of the 100) outcomes. Therefore, (5%/50%) of the decisions would be determined by the AI tool.

Scenario 2: You are contacted about an AI tool that forecasts which (team members/projects) will benefit the most from extra resources. The AI tool will choose which (team members/projects) to allocate resources to based on current organizational needs. When you read the memo about the AI tool, the software (provides/does not provide) the algorithm used to reach its decisions. This means you (would/would not) know what factors the AI tool uses to inform its decisions. If you decide to use this tool, it will make (3 out of 50/30 out of 50) decisions about allocating resources. Therefore, (6%/60%) of organizational funds will be allocated based on the AI tool's decisions.

Scenario 3: You receive an inquiry about an AI tool that rates (employee/product) performance based on measurable traits. The AI tool assigns a score to each trait of the (employee/product), and all trait scores will be added up to create a total score. When making a total score, the AI tool (tells/does not tell) you how it decides the importance of each trait. This means you would (know/not know) how much weight the AI tool gives to each trait in the final score. The AI tool would be responsible for producing (11 out of the 200/110 out of the 200) ratings. Therefore, (5.5%/55%) of the ratings would be determined by the AI tool.

Appendix B

Perceived Ethicality of AI Scale

(1= strongly disagree to 5=strongly agree)

The AI tool makes decisions in a clear and open manner

The AI tool will handle the data it collects in a way that protects privacy

There are parties responsible for the AI tool

The decisions from using this AI tool will be equitable

The AI tool is transparent

The AI tool seems to be developed in a way that avoids harm

There will be accountability for the decision output of the AI tool

The AI tool cannot protect privacy*

The AI tool is ethical

The outcomes from using this AI tool will be unbiased

The AI tool could be misused if implemented*

The AI tool discloses its technical processes

The AI tool is fair

The AI tool will handle the data it generates in a way that protects privacy

The AI tool minimizes harm

There will be accountability for the implementation and use of the AI tool

*Reverse Coded

Functionality Trust in AI

(1= strongly disagree to 5=strongly agree)

The AI tool works well

The AI tool has the features necessary to complete key tasks

The AI tool is competent in its area of expertise

The AI tool is dependable

The AI tool is reliable

Likelihood to Recommend the AI Tool

(1= extremely unlikely to 6=extremely likely, no neutral option)

How likely are you to recommend the use of this AI tool in your organization?
